# Associations between preconception macronutrient intake and birth weight across strata of maternal BMI

**DOI:** 10.1371/journal.pone.0243200

**Published:** 2020-12-02

**Authors:** Nastaran Salavati, Marian K. Bakker, Fraser Lewis, Petra C. Vinke, Farya Mubarik, JanJaap H. M. Erwich, Eline M. van der Beek

**Affiliations:** 1 Department of Obstetrics and Gynecology, University Medical Centre of Groningen, University of Groningen, Groningen, The Netherlands; 2 Department of Genetics, EUROCAT Registration Northern Netherlands, University Medical Centre of Groningen, University of Groningen, Groningen, The Netherlands; 3 Danone Nutricia Research, Utrecht, The Netherlands; 4 Department of Epidemiology, University Medical Centre of Groningen, University of Groningen, Groningen, The Netherlands; 5 Division of Human Nutrition, Wageningen University and Research Centre, Wageningen, The Netherlands; 6 Department of Pediatrics, University Medical Centre of Groningen, University of Groningen, Groningen, The Netherlands; University of Cape Coast, GHANA

## Abstract

**Introduction:**

Maternal nutrition during pregnancy is linked with birth outcomes including fetal growth, birth weight, congenital anomalies and long-term health through intra-uterine programming. However, a woman’s nutritional status before pregnancy is a strong determinant in early embryo-placental development, and subsequently outcomes for both mother and child. Therefore, the aim of this study was to investigate the association between dietary macronutrient intake in the preconception period with birth weight.

**Methods:**

We studied a group of 1698 women from the Dutch Perined-Lifelines linked birth cohort with reliable detailed information on preconception dietary macronutrient intake (using a semi quantitative food frequency questionnaire) and data available on birth weight of the offspring. Birth weight was converted into gestational age adjusted z-scores, and macronutrient intake was adjusted for total energy intake using the nutrient residual method. Preconception BMI was converted into cohort-based quintiles. Multivariable linear regression was performed, adjusted for other macronutrients and covariates.

**Results:**

Mean maternal age was 29.5 years (SD 3.9), preconception BMI: 24.7 kg/m^2^ (SD 4.2) and median daily energy intake was 1812 kcal (IQR 1544–2140). Mean birth weight was 3578 grams (SD 472). When adjusted for covariates, a significant association (adjusted z score [95% CI], P) between polysaccharides and birth weight was shown (0.08 [0.01–0.15], 0.03). When linear regression analyses were performed within cohort-based quintiles of maternal BMI, positive significant associations between total protein, animal protein, fat, total carbohydrates, mono-disaccharides and polysaccharides with birth weight were shown in the lowest quintile of BMI independent of energy intake, intake of other macronutrients and covariates.

**Conclusion:**

Out of all macronutrients studied, polysaccharides showed the strongest association with birth weight, independent of energy intake and other covariates. Our study might suggest that specifically in women with low preconception BMI a larger amount of macronutrient intake was associated with increased birth weight. We recommend that any dietary assessment and advise during preconception should be customized to preconception weight status of the women.

## Background

It has been widely acknowledged that adequate maternal nutrition, including maternal dietary intake before and during pregnancy, is a fundamental prerequisite for optimal growth, development and long term health of the offspring. Previous studies described deleterious effects of severe macronutrient deficiency on pregnancy outcome that depend on the stage of gestation [1, 2]. Worldwide, many women have a suboptimal nutrient status at the time of conception, which is also related to the fact that 4 out of 10 pregnancies are reported to be unplanned [3]. These unplanned pregnancies may have the highest risk of insufficient diets and inadequate nutrient intake.

Although severe under nutrition and extreme low energy intakes are not very common for pregnant women in the western world today, differences in the contribution of macronutrients to the total energy intake potentially are. Godfrey et al (1997) examined the effect of maternal diet during pregnancy on the ponderal index (measure for weight in relation to height) of the offspring in the Southampton Women Survey [4], (data collection between 1998–2002 [5]). They showed that high carbohydrate intake in early pregnancy, especially combined with low dairy protein intake in late pregnancy, was associated with a low ponderal index, meaning that these infants were thin at birth.

Recent studies suggest that maternal dietary intake in the preconception period can already play a vital role in early embryonic and placenta development and thus affect pregnancy outcomes, as various major organs are already formed during the first weeks of pregnancy [6–8]. Therefore, we emphasize that optimal maternal dietary intake is important before, as well as, during pregnancy. However, to date, very few observational studies have examined preconception diet in relation to birth weight. The studies that did, have limited sample sizes and/or focused on intake of a single macronutrient rather than more complete dietary patterns and macronutrient composition [9, 10].

Using data from the Perined-Lifelines linked birth cohort [11], we aimed to investigate the association between intakes of specific dietary macronutrients; i.e. protein, carbohydrate and fat, and their quality such as plant and animal protein, and mono-di and polysaccharides, with birth weight, in a well-nourished, representative sample of women of fertile age in a western Caucasian population, with the majority having a normal BMI according to the WHO definition [12]. We aimed to investigate this in both the complete cohort, as well as stratified groups of maternal preconception BMI.

Results from this study may contribute to the generation of more knowledge on the relationship between nutrition in the preconception period and pregnancy outcomes. With these insights, nutritional care for women of childbearing age can be further improved, aiming to optimize the health of both the women of childbearing age, as well as that of their (future) offspring.

## Materials and methods

This study is part of the Perined-Lifelines linked birth cohort, a cohort linked between the Dutch national birth registry (Perined) [13] and the Lifelines Cohort study [14].

### Overview of the Perined-Lifelines linked birth cohort

The Perined-Lifelines linked birth cohort was created by linking two existing databases; a large population-based cohort study (The Lifelines Cohort study, [14]) and the national birth registry (Perined, [13]), through a ‘trusted third party’ (‘ZorgTTP’ Houten, The Netherlands), facilitated by Mondriaan project (UMCG)/Lygature (Utrecht, The Netherlands) and has been described previously in detail [11]. Lifelines is a multi-disciplinary prospective population-based cohort study examining in a unique three-generation design the health and health-related behaviours of 167,729 persons living in the North of the Netherlands. It employs a broad range of investigative procedures in assessing the biomedical, socio-demographic, behavioural, physical and psychological factors which contribute to the health and disease of the general population, with a special focus on multi-morbidity and complex genetics. Female participants from the Lifelines Cohort study who indicated in their first or second follow-up questionnaire to have delivered a child since the previous questionnaire were selected. The information collected at baseline (e.g. demographical variables, detailed nutrient intake) was considered as the pre-conceptional information available for that specific pregnancy. Since the Lifelines Cohort Study does not collect information on pregnancy or pregnancy outcomes, the female participants from Lifelines were linked with the information on their pregnancy outcomes available via the national birth registry (Perined). This was done through corresponding pseudonyms in Lifelines and Perined, created based on three personal linking variables (birth date and 4-digits ZIP code of the residential address of the female participants from Lifelines, and birth date of their child). This resulted in a Perined-Lifelines linked birth cohort, containing information on dietary intake during the period prior to conception as well as pregnancy outcomes.

### Study group

Among the women in the Perined-Lifelines linked birth cohort, the inclusion criteria for the present analyses were delivery of a live born baby at term (≥ 37 weeks' gestational age) and availability of information on birth weight of their offspring. Women with unreliable data for dietary intake were excluded from the analyses. Reliability of reported dietary intake was based on the ratio of reported energy intake and basal metabolic rate [15, 16]; ratio below 0.50 or above 2.75 was considered as not reliable and excluded from further analysis. Also, intake of women with less than 500kcal/day was considered as unreliable reported dietary intake [17, 18].

### Maternal macronutrient intake

Macronutrient intake (i.e. total protein, animal, and vegetable protein, fat, total carbohydrates, mono- and disaccharides and polysaccharides) in the period prior to pregnancy was assessed with a 110 item semi-quantitative, food-frequency questionnaire (FFQ) [19] that women filled in at enrolment in The Lifelines Cohort study. The FFQ assessed food intake over the previous month. The average daily intake of the macronutrients and energy was calculated using the Dutch 2011 food composition table [20]. Maternal macronutrient intake was adjusted for total energy (based on reported dietary intake at the FFQ) using the nutrient residual method to evaluate the effect of maternal macronutrient intake independent of energy intake and to reduce the magnitude of the measurement error [17]. This approach produces a nutrient measure not correlated with energy intake. Subsequently, quintiles were generated by use of the distribution of the study population, whereby quintile 1 contained the 20% with the lowest consumption of that specific macronutrient, and quintile 5 the 20% highest intake.

### Maternal and fetal characteristics

Preconception maternal BMI was calculated based on measured height and weight at the Lifelines research sites at enrolment (baseline) to Lifelines. Height and body weight were measured without shoes and heavy clothing with the SECA 222 stadiometer and the SECA 761 scale. For the description of the cohort it was first grouped using the WHO classification: underweight (BMI < 18.5 kg/m^2^), normal weight (BMI 18.5–25.0 kg/m^2^), overweight (BMI > 25.0 kg/m^2^). To understand to what extent possible associations could be attributed to specific groups of BMI, and which BMI group may potentially benefit most from changes in lifestyle, BMI quintiles were generated by use of the distribution within the study, whereby quintile 1 was defined as ‘low’ BMI (lowest 20% in this cohort), quintiles 2 to 4 as ‘normal’ BMI (middle 60% of this cohort and used as the reference) and ‘high’ BMI was based on quintile 5 (20% highest BMI in this cohort). The maximum possible time between the FFQ and birth of the child is the time between the FFQ at baseline and the follow-up questionnaire where the women filled in they delivered a child since the previous questionnaire. Maternal age was age at enrollment/baseline in Lifelines. Maternal education was assigned in three categories: low (no education, primary school, lower vocational or lower general secondary education), intermediate (intermediate vocational training or higher secondary education) and high (higher vocational or university education) education. Maternal ethnicity was classified as either ‘white/European’ and ‘other’. Maternal smoking was divided into ‘smoking’ or ‘non-smoking’ as indicated at baseline. Maternal alcohol use was divided into ‘alcohol use’ (defined as alcohol use at moment of baseline/FFQ) and ‘no alcohol use’ [14]. Urbanisation level was categorized as ‘very high’, ‘high’, ‘moderate’, ‘low’, ‘rural’ based on the four-digit ZIP-code. Parity was categorized as one, two, or >/ = three. Birth weight was recorded in grams in Perined, and converted into a gestational age (GA)- adjusted z-score to adjust for variation in gestational age.

### Statistical methods

Continuous variables were summarized by the median and IQR, and comparisons between groups were made by the Kruskal-Wallis test. Distributions of categorical variables were compared using a Wilcoxon-type test for trend. The associations between preconception maternal macronutrient intake (exposure) and birth weight (z-scores; adjusted for gestational age) (outcome) were estimated by linear regression. Adjusted analyses were performed using multivariable linear regression, using different multivariable models (different covariates included). Based on the R-squared and Akaike Information Criteria (AIC), estimators of the relative quality of statistical models of a given set of data, the best model will be reported (the higher the R-squared, or the lower the AIC, the better the model). Least Absolute Shrinkage and Selection Operation (LASSO) regression analyses was performed to examine the strongest predictor of birth weight out of all the macronutrients and covariates [21]. LASSO identifies the strongest predictive variables and zeroes out the irrelevant ones by penalizing regression coefficients using regularization [21].

Linear regression analyses between macronutrient intake and birth weight were performed within the complete cohort. To test whether the association of macronutrients intake with birth weight is modified by maternal BMI, two different sets of analyses were conducted. Firstly, interaction terms between macronutrients intake and categorical BMI (cohort based quintiles) were included into the regression model within the complete cohort, and secondly separately regression models were fitted within each strata of BMI (cohort based quintiles). Statistical significance was assumed at P < 0.05. Analyses were performed in SPSS version 23 (IBM Corp., Armonk, NJ, USA).

## Results

### Description of the study population

**Fig 1:**
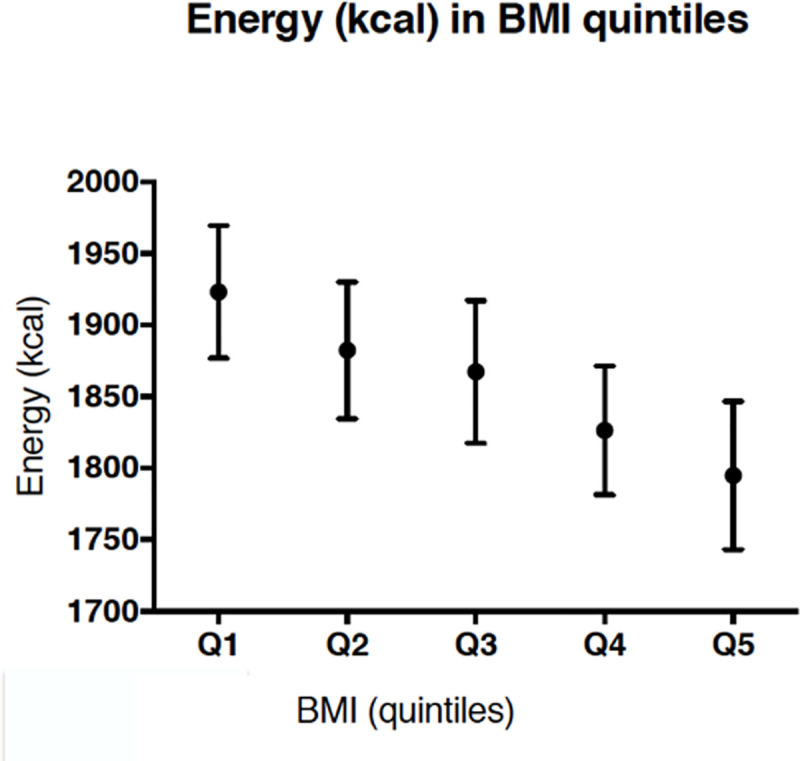
Mean energy intake (kcal) in BMI quintiles (95% confidence intervals).

A total of 2,368 women from The Lifelines Cohort Study could be linked to available data in Perined. After excluding women who did not have reliable, or missing dietary intake reported (resp. n = 427 and n = 168), pre-term births (gestational age <37 weeks; n = 110) and unknown sex of the child (n = 1), 1,698 women remained available for analyses. The characteristics of the study cohort, presented as three groups of BMI; ‘low’ BMI (quintile 1; n = 329), ‘normal’ BMI (quintiles 2–4; n = 1043), and ‘high’ BMI (quintile 5; n = 326), are summarised in Table 1. The lower BMI group is relatively higher educated, has a lower urbanisation level and the percentage of alcohol users is slightly higher compared to the high BMI group. In addition, the percentage of nulliparous women is higher in the low BMI group (Table 1). The birth weight also increases over BMI groups (Table 1). Furthermore, the intake of energy showed a small but consistent decrease over BMI quintiles (Kendalls tau correlation coefficient = -0.079; p <0.001) (Fig 1). Linear regression analyses between energy intake and BMI showed a (weak) negative association (β = -0.001, p<0.001), with R-squared = 0.007 and AIC = 4863.

**Table 1:** Characteristics of the cohort according to the quintile of maternal BMI.

Characteristics	Low BMI (Q1)^1^ N = 329 (100%)		Normal BMI (Q2-Q4)^1^ N = 1043 (100%)		High BMI (Q5)^1^ N = 326 (100%)		P*
**Demographics**							
Age at enrollment in years	29	(27–32)	29	(27–32)	29	(27–33)	0.36
Ethnicity							0.55
*White*, *East/West European Ethnicity*	323	(98.2)	1015	(97.3)	323	(99.1)	
*Other*	6	(1.8)	28	(2.7)	3	(0.9)	
Education^2^							<0.001
*Low*	12	(3.7)	58	(5.6)	35	(10.9)	
*Moderate*	99	(30.7)	397	(38.4)	141	(43.8)	
*High*	212	(65.6)	578	(56.0)	146	(45.3)	
Missing	6		10			4	
Urbanization level by category ^3^							0.002
*1*	93	(28.5)	249	(24.5)	61	(18.9)	
*2*	23	(7.1)	83	(8.2)	27	(8.4)	
*3*	24	(7.4)	69	(6.8)	25	(7.8)	
*4*	53	(16.3)	111	(10.9)	36	(11.2)	
*5*	133	(40.8)	506	(49.7)	173	(53.7)	
Missing	3		25			4	
**Diet**							
Energy intake (kcal/day)	1898	(1624–2202)	1802	(1545–2129)	1781	(1482–2072)	<0.001
Percentage energy from: ^4^							
*Carbohydrates*	47.2	(44.4–50.5)	46.4	(43.4–49.8)	46.4	(43.0–49.5)	0.01
*Mono and Di saccharides*	24.7	(21.5–29.0)	24.5	(21.1–28.5)	24.6	(21.0–28.8)	0.80
*Polysaccharides*	29.9	(26.8–32.7)	29.1	(26.4–31.8)	28.7	(26.0–31.7)	0.02
*Protein*	14.1	(13.1–15.4)	14.6	(13.4–16.0)	15.3	(13.8–16.6)	<0.001
*Animal protein*	8.0	(6.6–9.2)	8.5	(7.3–9.9)	9.2	(8.1–10.7)	<0.001
*Plant protein*	6.1	(5.7–6.9)	6.0	(5.4–6.7)	5.9	(5.4–6.5)	<0.001
*Fat*	34.9	(31.7–37.9)	34.8	(31.9–37.8)	34.9	(32.1–38.1)	0.84
**Lifestyle**							
BMI ^5^ (kg/m^2^)	20.3	(19.6–20.8)	23.8	(22.6–25.5)	30.5	(28.9–32.9)	<0.001
BMI WHO classification							<0.001
*<18*.*5*	19	(5.8)	0			0	
*18*.*5-<25*	310	(94.2)	709	(68.0)		0	
*25-<30*	0		334	(32.0)	135	(41.4)	
*≥ 30*	0		0		191	(58.6)	
Alcohol							
*User percentage (%)*	261	(79.3)	830	(79.7)	226	(69.3)	0.001
*Median consumption* ^6^ (g/day)	2.3	(0.8–6.1)	2.5	(1.2–5.8)	1.5	(0.6–3.9)	<0.001
*Missing*	0		2			0	
Smoker	38	(11.6)	131	(12.6)	43	(13.3)	0.65
*Missing*	0		1			2	
**Pregnancy**							
Maximum time between baseline questionnaire and birth child (in months)	12.0	(11.0–15.0)	13.0	(11.0–16.0)	13.0	(11.0–16.0)	0.27
Sex of the child							0.79
*Male*	165	(50.2)	520	(49.9)	164	(50.3)	
*Female*	164	(49.8)	523	(50.1)	162	(49.7)	
Gravidity							0.02
*1*	150	(45.6)	422	(40.5)	120	(36.8)	
*2*	107	(32.4)	352	(33.7)	119	(36.5)	
*3*	46	(14.0)	169	(16.2)	60	(18.4)	
*≥4*	26	(7.9)	100	(9.6)	27	(8.3)	
Parity							0.002
*0*	181	(55.0)	494	(47.4)	138	(42.3)	
*1*	109	(33.1)	384	(36.8)	139	(42.6)	
*≥2*	39	(11.9)	165	(15.8)	49	(15.0)	
Birth weight (in grams)	3410	(3110–3760)	3598	(3280–3890)	3640	(3267–3988)	<0.001
*Missing*	2		1			1	
Gestational age (in weeks)	39.0	(39.0–40.0)	39.0	(39.0–40.0)	38.0	(40.0–40.0)	0.36
Apgar-score (after 5 min)							0.06
*<10*	73	(22.3)	257	(24.6)	92	(28.2)	
*10*	254	(77.7)	786	(75.4)	234	(71.8)	
*Missing*	2						

Data are median (IQR) or n (%). Data were complete when there is no missing row presented.

^1^Q1 = Quintile 1 ranging from from 17.1–21.2 kg/m^2^, Q2-Q4 = Quintiles 2–4 ranging from 21.3–27.5 kg/m^2^, Q5 = Quintile 5 ranging from 27.6–47.3 kg/m^2^.

^2^Low education: primary school, vocational and lower general secondary education; Moderate education: higher secondary education and intermediate vocational training; High education: higher vocational education and university education.

^3^Level of urbanization: 1. Very high > = 2500 addresses per km^2^; 2: high 1500–2500 addresses per km^2^; 3: moderate 1000–1500 addresses per km^2^; 4: low 500–1000 addresses per km^2^; 5: rural <500 addresses per km^2^.

^4^Energy from carbohydrates, protein and fat, relative to the sum of energy from the three macronutrients

^5^BMI = Body mass index

^6^Median + IQR among alcohol users. One standard drink contains 10 g alcohol.

*Two sided p value Kruskal Wallis for continuous characteristics or Wilcoxon-type test for trend for categorical characteristics [22].

As shown in Table 1, the maximum possible time between FFQ and birth of the child was not significantly different among the BMI quintiles. When the average pregnancy duration of 9 months was subtracted from this period, it was shown that within the complete cohort, 48.0% of the women filled in the FFQ within 0–3 months before the start of the pregnancy. For 26.4% of the women this maximum period was 4–6 months, for 8.3% of the women between 7–9 months, for 6.5% of the women between 10–12 months, and 10.8% of the women had a maximum time of more than 12 months between the FFQ and pregnancy.

When characteristics of the cohort were compared between groups of BMI using the WHO classification, the results were in line with results reported in Table 1.

The study cohort was representative in terms of diet quality in comparison with the complete Lifelines Cohort study [11, 23]. The characteristics of dietary intake with respect to macronutrients, are summarised in Table 1.

### Regression analysis results- complete cohort

**Fig 2:**
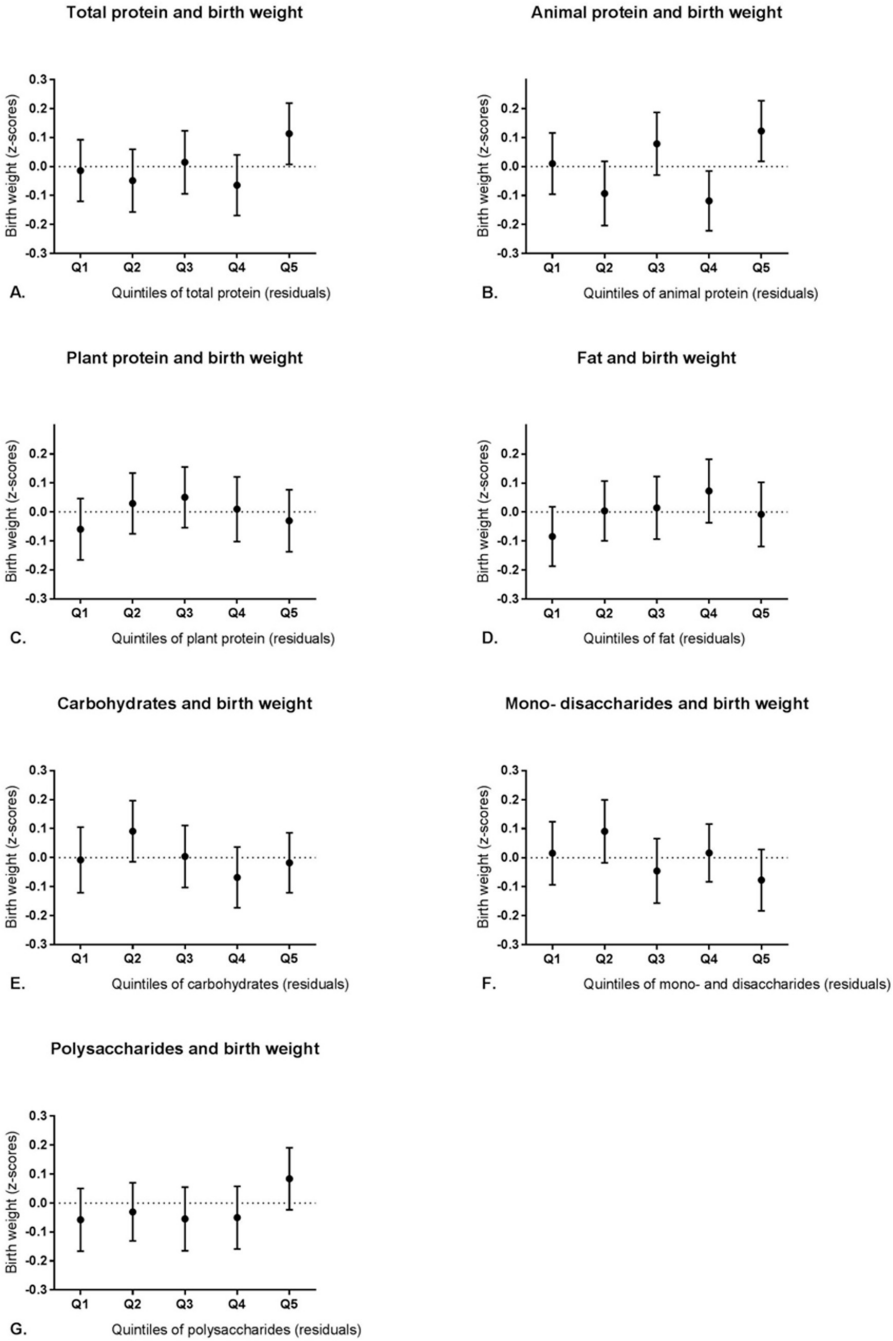
Mean birth weight (z-scores; adjusted for gestational age) in quintiles of macronutrient intake (95% confidence intervals). A. Total protein, B. animal protein, C. plant protein, D. fat, E. carbohydrates, F. mono- and disaccharides, G. polysaccharides.

A range of linear regression models with different combinations of covariates were considered to investigate which model showed the best explaining variance of birth weight outcome. The model with the best goodness of fit (based on the R-squared and AIC) was the model with adjustment for intake of other macronutrients, maternal BMI, maternal age, smoking, alcohol, education level, urbanization level, parity, sex of newborn, ethnicity and energy intake (in kcal) (R-squared = 0.12; AIC = -182.42). Linear regression analysis within this model showed that increased intake of polysaccharides was associated with increased birth weight (adjusted z-score = 0.076 [95% CI 0.001 to 0.144, p = 0.03]) (Table 2). In addition, birth weight mainly increased in the highest quintile of polysaccharides intake (Fig 2G). The results of all other variables (i.e. covariates and macronutrients) included in the model can be found in supporting information (S1 Table).

**Table 2:** Linear regression analysis of macronutrient intake in relation to birth weight (n = 1698, 100%).

	Linear regression analysis^1^	
**Analysis**	Coeff (95% CI)^2^	P
Total protein	0.020 (-0.056–0.096)	0.61
Animal protein	0.020 (-0.062–0.103)	0.63
Plant protein	0.028 (-0.035–0.090)	0.39
Fat	0.019 (-0.027–0.065)	0.41
Total carbohydrates	0.045 (-0.109–0.20)	0.55
Mono- and disaccharides	0.030 (-0.058–0.12)	0.51
Polysaccharides	**0.076 (0.008–0.144)**	**0.03**

^1^Adjusted for intake of other macronutrients, maternal BMI, maternal age, smoking, alcohol, education level, urbanization level, parity, sex of newborn, ethnicity, energy intake (in kcal)

^2^ Coefficients are expressed as z-scores, i.e. the unit for the coefficients is one standard deviation (SD).

Additional analyses were performed with possible outliers of BMI (n = 29) excluded, however did this not affect the results. Therefore, the results showed here are with these cases included.

### Lasso regression analysis

Lasso regression analysis was performed including all macronutrients, covariates and birth weight, to check which variables are the strongest predictor for birth weight. No robust statistical associations were found between macronutrients and birth weight. However, lasso regression models exhibited a robust statistical association between maternal preconception BMI (adjusted z-score = 0.095; p<0.001), parity (adjusted z-score = 0.145; p<0.001) and sex of the child (adjusted z-score = -0.084; p<0.001) with birth weight, with maternal BMI being the only modifiable factor.

### Regression analysis results- in quintiles of BMI

To investigate if the association between preconception macronutrient intake with birth weight can be attributed to specific groups of BMI, linear regression analyses were performed within cohort based BMI quintiles (adjusted for intake of other macronutrients, maternal BMI, maternal age, smoking, alcohol, education level, urbanization level, parity, sex of newborn, ethnicity). These analyses showed that within the group of women with the lowest BMI (min, max BMI: 17.1–21.2), there was a significant positive association between animal protein, fat, total carbohydrates, mono-and disaccharides and polysaccharides and birth weight (Table 3). Additional adjustment for energy intake (in kcal) did not change these results. The interaction terms between each specific macronutrient and BMI in quintiles were not significant (Table 3).

**Table 3:** Linear regression analysis of macronutrient intake in relation to birth weight in quintiles of BMI (n = 1698, 100%).

	Coeff (95% CI)^1^ for birth weight (z-scores)					
	BMI quintiles^2^					
	Q1 (n = 329)	Q2 (n = 345)	Q3 (n = 347)	Q4 (n = 337)	Q5 (n = 340)	P^5^
Energy (in kcal)^3^	5.12E^-^^5^ (0.00 to 0.00)	1.68E^-^^5^ (0.00 to 0.00)	6.66E^-6^ (0.00 to 0.00)	-2.28E^-^^5^ (0.00 to 0.00)	-5.36E^-^^5^ (0.00 to 0.00)	0.86
Total protein^4^	**0.19 (0.01 to 0.37)**	-0.13 (-0.29 to 0.02)	-0.04 (-0.20 to 0.12)	0.14 (-0.05 to 0.32)	0.04 (-0.16 to 0.24)	0.49
Animal protein^4^	**0.21 (0.01 to 0.40)**	-0.14 (-0.31 to 0.02)	-0.05 (-0.23 to 0.12)	0.15 (-0.05 to 0.35)	0.02 (-0.20 to 0.23)	0.90
Plant protein^4^	0.08 (-0.05 to 0.22)	-0.08 (-0.22 to 0.06)	0.02 (-0.11 to 0.14)	0.08 (-0.09 to 0.25)	0.15 (-0.008 to 0.31)	0.12
Fat^4^	**0.37 (0.03 to 0.72)**	-0.04 (-0.33 to 0.24)	-0.08 (-0.37 to 0.21)	0.33 (-0.06 to 0.72)	-0.002 (-0.36 to 0.36)	0.78
Total carbohydrates^4^	**0.47 (0.08 to 0.86)**	-0.09 (-0.40 to 0.22)	-0.17 (-0.49 to 0.15)	0.39 (-0.02 to 0.79)	-0.05 (-0.43 to 0.32)	0.45
Mono- and disaccharides^4^	**0.19 (-0.002 to 0.39)**	-0.09 (-0.29 to 0.11)	-0.05 (-0.23 to 0.14)	0.11 (-0.09 to 0.31)	-0.03 (-0.25 to 0.19)	0.82
Poly-Saccharides^4^	**0.20 (0.05 to 0.35)**	0.01 (-0.15 to 0.17)	0.07 (-0.08 to 0.22)	0.04 (-0.12 to 0.20)	0.05 (-0.11 to 0.21)	0.41

^1^Coefficients are expressed as z-scores, i.e. the unit for the coefficients is one standard deviation (SD).

^2^Quintile 1, ranging from 17.1–21.2 kg/m^2^ (n = 329), Q2 = Quintile 2, ranging from 21.3–22.9 kg/m^2^ (n = 345), Q3 = Quintile 3, ranging from 23.0–24.8 kg/m^2^ (n = 347), Q4 = Quintile 4, ranging from 24.9–27.5 kg/m^2^ (n = 337), Q5 = Quintile 5, ranging from 27.6–47.3 kg/m^2^ (n = 340).

^3^Adjusted for maternal BMI, maternal age, smoking, alcohol, education level, urbanization level, parity, sex of newborn, ethnicity.

^4^Adjusted for intake of other macronutrients*, maternal BMI, maternal age, smoking, alcohol, education level, urbanization level, parity, sex of newborn, ethnicity.

^5^P for interaction.

*Model for: total protein (adjustment with fat, total carbohydrates), animal protein (adjustment with plant protein, fat, total carbohydrates), plant protein (adjustment with animal protein, fat, carbohydrates), fat (adjustment with total protein, total carbohydrates), total carbohydrates (adjustment with total protein, fat), mono- and disaccharides (adjustment with total protein, fat, poly-saccharides), poly-saccharides (adjustment with total protein, fat, mono- and disaccharides).

Associations in bold are significant at p < 0.05.

### Birth weight in BMI quintiles

**Fig 3:**
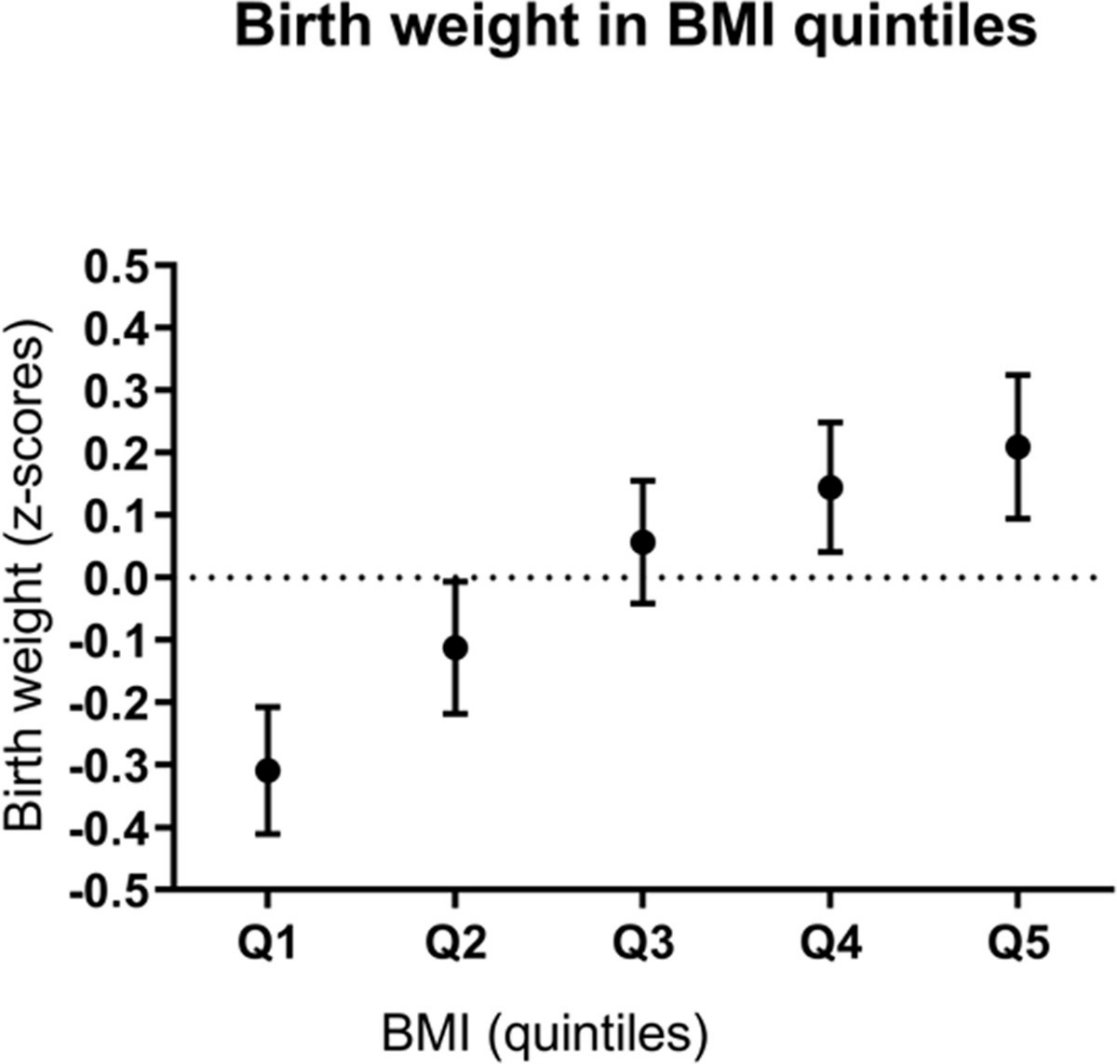
Mean birth weight (z-scores; adjusted for gestational age) in BMI quintiles (95% confidence intervals).

Linear regression analyses within quintiles of BMI showed consistent positive significant associations between specific macronutrients intake and birth weight. As shown in Fig 3, BMI quintile 1 is also the quintile with the lowest mean birth weight of the offspring, different from the increasing birth weight trend over the remaining BMI quintiles. To investigate whether the association found in BMI quintile 1 was possible driven by the fact that these are the children with the lowest birth weight, adjusted linear regression analyses between macronutrient intake and birth weight, was performed within cohort base birth weight quintiles. No significant association between any of the macronutrients and birth weight in the birth weight quintiles was found.

## Discussion

The aim of this study was to investigate the relationship between maternal macronutrient intake and birth weight of the offspring in a representative group of women from fertile age in a western Caucasian population, with the majority having a normal BMI according to the WHO definition [12].

To the best of our knowledge, this is the first large cohort study to explore the relationship between maternal dietary macronutrient intake in the preconception period and birth weight of the offspring. Within this (relatively) healthy, homogenous population, with minimal variation in adjusted birth weight, we observed that out of all the macronutrients studied, preconception intake of polysaccharides appears to have the strongest association with birth weight, independent of energy intake and maternal characteristics. However, in line with other studies, maternal BMI, parity and sex of the child showed a more robust association with birth weight than maternal intake of macronutrients.

We evaluated the association of maternal macronutrient intake with offspring’s birth weight for specific groups of BMI and found that within the group of women with a lower preconception BMI, higher macronutrient intake (except for plant protein) was associated with an increased birth weight, independent of energy intake and maternal characteristics. However, the interaction term between BMI quintiles and macronutrient intake was not significant. An association between total energy intake (in kcal) and birth weight was not shown in this cohort, both when analyses were performed in the complete cohort as in BMI quintiles. Therefore, the association between an increase of specific macronutrients intake and birth weight was not explained by total energy intake.

We showed that higher maternal intake of polysaccharides during preconception was associated with higher birth weight of the offspring. This finding is in agreement with a study which investigated this association during pregnancy [24]. Sharma et al. observed that higher intake of polysaccharides (starch) was associated with increased odds of delivering LGA infants [24]. Another study compared dietary intake of women with normal pregnancies versus women with gestational diabetes mellitus (GDM), and found that women who consumed a carbohydrate-rich diet were likely to have high blood glucose levels, and therefore had an increased risk of delivery LGA offspring [25]. A possible explanation for these results could be that higher intake of carbohydrates could lower maternal insulin sensitivity, making higher levels of free glucose available for placental circulation, subsequently activating fetal glycogenesis [26]. Despite the fact that we found an association between polysaccharides and birth weight, we did not find any association between total carbohydrates, mono- and disaccharides and birth weight. Mono- and disaccharides and polysaccharides may have different metabolic effects on postprandial blood glucose levels due to their digestibility and structure. To explain how different kinds of carbohydrate-rich foods directly affect blood sugar, the glycemic index was developed and is considered an appropriate way of categorizing carbohydrates and its effect on blood glucose levels. High-glycemic-index foods, which causes powerful spikes in blood sugar, can lead to an increased risk for type 2 diabetes [27], heart disease [28], and overweight [29–31]. In contrast, low-glycemic index diets, which causes slower blood sugar rises, may offer anti-inflammatory benefits [32]. To elucidate the possible impact of different types of carbohydrates during preconception, further research of food groups distinguished by glycemic index, and their association with birth weight, needs to be conducted.

We did not find any significant association between maternal intake of the other macronutrients and birth weight when we performed analysis within the complete cohort. Our study participants were adequately nourished [33] and there was minimal variation of the adjusted birth weight of the offspring. This resulted in a relative narrow distribution of both determinant and outcome, hence this might be the reason we did not notice any effect for other macronutrients. Despite the fact that we did not see a significant association between protein, including plant protein, and birth weight, we found an ‘U-shaped’ association with birth weight. Both low and high intake of plant protein showed slightly lower mean birth weights.

These observations are in line with the study from Switkowski et al. who also reported such a ‘U-shaped’ association with decreasing birth weight upon increments in protein intake among a group of pregnant women (n = 1961) [34]. In addition, this ‘U-shaped’ association has also been previously observed by protein intake during pregnancy by Sloan et al. (2001) [35]. Women with either high (>85 grams) or low (<50 grams) protein intake had babies with lower birth weight. These levels of protein intake were not very common in our cohort.

Although studies investigating macronutrient intake during preconception and birth weight are scarce, several epidemiological studies showed an association between dietary intake during pregnancy and birth weight. However, contradictory results have been reported. On the one hand, Haste et al. [36], Godfrey et al. [37] and Cuco et al. [9], found that maternal nutrition during pregnancy had an important effect on birth weight. For example, the study from Cuco et al. described a positive association between protein intake during preconception and birth weight [9]. Yet Mathews et al [38] and Lagiou et al. [39] reported no effects.

In order to investigate which of the variables (macronutrients intake and maternal characteristics) were the strongest predictor of birth weight, we performed LASSO regression analysis. Maternal BMI, parity and sex of the child appeared to be the strongest predictors of birth weight within our study. This finding is in line with a study from Radesky et al., who investigated the association between nutrients and dietary patterns with the risk of gestational diabetes mellitus, and showed that pre-pregnancy BMI might be of greater importance than the maternal diet for the development of gestational diabetes [40]. Individually macronutrient intake on itself is probably less informative than pre-pregnancy BMI as this is a representative measurement of not only dietary intake, but also dietary/lifestyle behaviour and physical activity. Out of the three variables found via Lasso regression, maternal BMI is the only modifiable factor and is subject of several lifestyle intervention studies for women during preconception and pregnancy [41–45]. Therefore, we performed subsequent analyses in cohort based BMI quintiles to understand to what extent possible associations could be attributed to specific groups of BMI, and also which group may potentially benefit the most from changes in diet and lifestyle. The distribution of maternal BMI is displayed in Table 1. According to the WHO classification, 60% in our cohort has normal BMI, 27.6% is overweight, 11.2% obese and 1.2% is underweight. More specifically, in the lowest BMI quintile there are not only women included with underweight according to the WHO, but also with a normal BMI. Since we have adjusted for maternal BMI within linear regression analyses, we do not expect this BMI distribution over BMI quintiles, to have influenced our results.

Adjusted linear regression showed a positive significant association between all the macronutrients (except plant protein) independently and birth weight in the lowest quintile of BMI (20% lowest BMI within our cohort). Although our results showed that the interaction term between specific macronutrients intake and birth weight in quintiles of BMI was not significant, we do not think this completely invalidates our findings. Other studies have found similar results, showing stronger associations between fruit and vegetable intake with increased birth weight among lean pregnant women [46]. The same pattern was reported in an Indian study [47], showing a stronger association between intake of green leavy vegetables and birth weight in the leanest women. Neggers et al., found an association between zinc and aspirin supplementation with birth weight only among women with low pre-pregnant BMI, and not among normal weight women [48]. They suggest that these associations, within the group of women with low BMI, may be mediated by a low plasma volume rather than by energy status. Rosso et al. [49], described that in underweight women, a low plasma volume during early pregnancy will result in proportionately reduced cardiac output. A lower cardiac output results in a lower uteroplacental blood flow and therefore decrease in transfer of nutrients to the fetus and consequently a possible reduction in fetal growth. It is suggested that within this group of women with low BMI, micro- and macronutrient intake is associated with increased plasma volume, which may result in increased birth weight.

The fact that we only find and association in the lowest quintile of BMI, and not in the other quintiles, without the interaction term being significant, could potentially be due to the fact that women in the lowest quintile differ in terms of demographical information where we have not (been able to) adjusted for. Characteristics with a yet unknown epidemiological or biological influence can potentially explain the differences found between the BMI quintiles. In addition, although less likely, it could be due to the fact that the BMI quintiles are not randomized and so perhaps the lowest BMI quintile has the highest heterogeneity/variation in variables. Future studies that will focus on dietary intake in the preconception period, should pay attention to different groups of maternal BMI, also to those having low BMI within the preconception period.

This study has several strengths. This is a large cohort comprising of 1698 women containing detailed reliable dietary data from their preconception period and pregnancy outcome of their offspring. Dietary intake was assessed using a food frequency questionnaire, allowing detailed information about food types and amounts to be recorded without influencing the participant’s eating behaviour, decreasing chance of bias. Additionally, in order to minimize the confounding effect of how maternal intake affects birth weight, we adjusted for maternal age, preconception BMI, sex of the child, parity, gravidity, smoking, alcohol intake [23, 50–54]. As presented previously our cohort is representative in terms of dietary intake [33]. The complete Lifelines Cohort has been examined in terms of representativeness compared to the Dutch population in the Northern Netherlands, and the Lifelines Cohort showed a good overall representativeness [55]. To illustrate, energy intake in this cohort is comparable with the complete Lifelines population ages between 20–40 years [23]. Although the level of education was slightly higher in our cohort compared to all women in the Lifelines Cohort (age 20–40 years) [23], we do not expect this to have influenced any of our results, mainly because we have adjusted for level of education. In this study, only women with reliable dietary intake were included, as described in the methods section. It was shown previously that there were some differences in terms of demographic variables between women with reliable versus unreliable dietary intake [11]. Women with reliable dietary intake were more often higher educated, slightly younger at preconception, percentage of alcohol consumers was higher, and percentage of smokers was lower. These differences were considered logical and selection bias is not expected to play a role. In addition, women with reliable dietary intake were more often nulliparous, and consequently had a slightly lower mean birth weight (3570 vs 3640 grams) but this is not considered as a clinical relevant difference.

The maximum time between dietary assessment and conception among the women in our cohort is very short making it less plausible that dietary intake has changed in between these two time points. Besides this, diet tends to be quite stable over time [56], and changes in dietary habits after conception tend to be modest and mostly reflect intake before conception [57].

There are few limitations, relevant to any study that explores dietary intake. The data in this cohort were not adjusted for maternal weight gain in pregnancy, since we only have weight of the mother during the preconception period and not during or after pregnancy. It is known that weight gain during pregnancy is associated with birth weight, and it may confound the association with maternal BMI. Therefore, the effect that we have found in this study could be related, at least in part, to differences in maternal weight gain.

Information on placental weight was unfortunately not available in our cohort. Together with birth weight it can give valuable information on potential growth restriction or risk for adverse outcomes for the offspring [58]. In the Dutch famine, dietary restriction during early gestation decreased the birth weight placental weight (BWPW)-ratio, and resulted in much greater risk of adult and coronary heart disease and obesity [59]. In future research focusing on preconception dietary intake, we suggest to investigate its association with the BWPW-ratio to distinguish newborns with a higher risk of adverse outcomes later in life [58, 59]. In addition, in future it would be interesting to perform additional analysis within specific sub-groups (e.g. small for gestational age, appropriate for gestational age, large for gestational age). These analyses have not been performed as this study is underpowered for this and consequently draw meaningful conclusions. Additionally, in future additional (diagnostic) parameters (e.g. ultrasound measurements) can be included by linking to other existing databases to describe more adverse pregnancy outcomes including pre-eclampsia and fetal growth restriction.

Our study included mostly women of Caucasian ethnicity, which limits extrapolation of the results to other ethnicities. However, the homogeneity of the study population makes the risk of possible confounding less likely.

When analyses were performed within cohort-based birth weight quintiles, no statistically significant association was shown between macronutrients intake and birth weight. This could be due to several reasons. First, from an epidemiologically point of view, maternal BMI is potentially more informative, and is having a stronger association with macronutrients, rather than birth weight on its own. From a statistical point of view, the distribution of birth weight is lower in the birth weight quintiles compared to distribution of birth weight in the BMI quintiles, making it more difficult to find a significant association.

Although we showed a negative association between preconception energy intake and maternal BMI, we consider this association very weak. The effect size was very low, with a R-squared/AIC close to zero and thus not likely to affect our results. The fact that women with the highest BMI had the lowest energy intake based on the FFQ, may be due to underreporting which has been described in literature before [60].

The primary advantage of representing diet as macronutrients is that such information can be directly related to our fundamental knowledge of biology [61]. Calculation of the total intake of a macronutrient (as opposed to using the contribution of a specific food-item or food group at a time) provides the most powerful test of a hypothesis, particularly if many foods each contribute modestly to intake of that nutrient. However, given the strengths and weaknesses of using nutrients or food items/groups to represent diet, it appears that an optimal approach would employ both. The case of causality is strengthened when an association is observed with overall intake of a nutrient and also with several food sources of that nutrient, particularly when the food sources are otherwise different. This provides, in some sense, multiple assessments of the potential for confounding by other nutrient; if an association as observed for only one food source of the nutrient, other factors contained in that food would tend to be similarly associated with the outcome/disease. Therefore, in future research association between food groups and birth weight needs to be further investigated.

## Conclusions

In conclusion, to the best of our knowledge, this is a first large study investigating dietary macronutrient intake in the preconception phase and its association with pregnancy outcome in a homogenous cohort. Out of all the macronutrients studied, polysaccharides showed the strongest association with birth weight, independent of energy intake and covariates.

This study underlines the importance of investigating dietary intake in the preconception phase and its association with pregnancy outcome. We recommend that future studies should focus on dietary intake in the preconception period, whereby different groups of women according to their preconception BMI need be made for analyses. With this, in future, dietary assessment and advise during the preconception phase can be tailored to weight status of the mother.

## Supporting information

S1 Table Linear regression analysis of macronutrient intake (adjusted for kcal) in relation to birth weight (n = 1698, 100%) (all terms from the model presented). ^1^Adjusted for intake of other macronutrients, maternal BMI, maternal age, smoking, alcohol, education level, urbanization level, parity, sex of newborn, ethnicity. ^2^ Coefficients are expressed as z-scores, i.e. the unit for the coefficients is one standard deviation (SD).
(DOCX)
