# Supplementary material for: Associations between preconception macronutrient intake and birth weight across strata of maternal BMI
Source: PLoS One. 2020 Dec 2;15(12):e0243200. doi: 10.1371/journal.pone.0243200 (PMC7710031; doi:10.1371/journal.pone.0243200)
Supplement: S1 Table — 1Adjusted for intake of other macronutrients, maternal BMI, maternal age, smoking, alcohol, education level, urbanization level, parity, sex of newborn, ethnicity. 2 Coefficients are expressed as z-scores, i.e. the unit for the coefficients is one standard deviation (SD). (DOCX) [file pone.0243200.s001.docx]

**Associations between preconception macronutrient intake and birth weight across strata of maternal BMI.**

**Analysis in the Dutch Perined-Lifelines birth cohort.**

Nastaran Salavati

Marian K. Bakker

Fraser Lewis

Petra C. Vinke

Farya Mubarik

JanJaap H.M. Erwich

Eline M. van der Beek

**S1 Table. Linear regression analysis of macronutrient intake (adjusted for kcal) in relation to birth weight (n=1698, 100%) (all terms from the model presented)**

|  | **Linear regression analysis^1^** | |
| --- | --- | --- |
| **Analysis** | Coeff (95% CI)^2^ | P |
| **Total protein** | 0.020  (-0.056- 0.096) | 0.61 |
| *Parity* | *0.28 (0.22-0,34)* | *<0.001* |
| *Maternal BMI* | *0.04 (0.02-0.05)* | *<0.001* |
| *Maternal age* | *-0.007 (-0.02-0.006)* | *0.30* |
| *Alcohol user* | *-0.07 (-0.19-0.05)* | *0.25* |
| *Smoking* | *0.20 (0.062-0.35)* | *0.005* |
| *Sex of the child* | *-0.35 (-0.44-0.26)* | *<0.001* |
| *Maternal ethnicity* | *-0.03 (-0.06-0.008)* | *0.13* |
| *Education* | *0.09 (0.006-0.16)* | *0.04* |
| *Urbanization level* | *0.009 (-0.02-0.04)* | *0.52* |
| *Fat (adj. for kcal)* | *0.06 (-0.08-0.20)* | *0.42* |
| *Total carbohydrates (adj. for kcal)* | *0.05 (-0.11-0.20)* | *0.56* |
|  |  |  |
| **Animal protein** | 0.020  (-0.062 – 0.103) | 0.63 |
| *Parity* | *0.28 (0.22-0.34)* | *<0.001* |
| *Maternal BMI* | *0.04 (0.03-0.05)* | *<0.001* |
| *Maternal age* | *-0.007 (-0.02 – 0.006)* | *0.27* |
| *Alcohol user* | *-0.07 (-0.19 – 0.05)* | *0.25* |
| *Smoking* | *0.20 (0.06-0.34)* | *0.005* |
| *Sex of the child* | *-0.35 (-0.44- -0.26)* | *<0.001* |
| *Maternal ethnicity* | *-0.03 (-0.06-0.008)* | *0.13* |
| *Education* | *0.08 (0.003-0.16)* | *0.04* |
| *Urbanization level* | *0.01 (-0.02 – 0.04)* | *0.49* |
| *Fat (adj. for kcal)* | *0.06 (-0.08-0.21)* | *0.38* |
| *Total carbohydrates (adj. for kcal)* | *0.05 (-0.11-0.20)* | *0.54* |
| *Plant protein (adj. for kcal)* | *0.03 (-0.04 – 0.09)* | *0.39* |
|  |  |  |
| **Plant protein** | 0.028  (-0.035 – 0.090) | 0.39 |
| *Parity* | *0.28 (0.22-0.34)* | *<0.001* |
| *Maternal BMI* | *0.04 (0.03-0.05)* | *<0.001* |
| *Maternal age* | *-0.007 (-0.02 – 0.006)* | *0.27* |
| *Alcohol user* | *-0.07 (-0.19 – 0.05)* | *0.25* |
| *Smoking* | *0.20 (0.06-0.34)* | *0.005* |
| *Sex of the child* | *-0.35 (-0.44 - -0.26)* | *<0.001* |
| *Maternal ethnicity* | *-0.03 (-0.06 – 0.008)* | *0.13* |
| *Education* | *0.08 (0.003-0.16)* | *0.04* |
| *Urbanization level* | *0.01 (-0.02 – 0.04)* | *0.49* |
| *Fat (adj. for kcal)* | *0.06 (-0.08-0.21)* | *0.38* |
| *Total carbohydrates (adj. for kcal)* | *0.05 (-0.11-0.20)* | *0.54* |
| *Animal protein (adj. for kcal)* | *0.02 (-0.06-0.10)* | *0.63* |
|  |  |  |
| **Fat** | 0.019  (-0.027 – 0.065) | 0.42 |
| *Parity* | *0.28 (0.22-0.34)* | *<0.001* |
| *Maternal BMI* | *0.04 (0.02-0.05)* | *<0.001* |
| *Maternal age* | *-0.007 (-0.02-0.006)* | *0.30* |
| *Alcohol user* | *-0.07 (-0.19 – 0.05)* | *0.25* |
| *Smoking* | *0.20 (0.06-0.35)* | *0.005* |
| *Sex of the child* | *-0.35 (-0.44- -0.26)* | *<0.001* |
| *Maternal ethnicity* | *-0.03 (-0.06 – 0.008)* | *0.13* |
| *Education* | *0.09 (0.006-0.16)* | *0.04* |
| *Urbanization level* | *0.009 (-0.02-0.04)* | *0.52* |
| *Total carbohydrates (adj. for kcal)* | *0.05 (-0.11 – 0.20)* | *0.56* |
| *Total protein (adj. for kcal)* | *0.02 (-0.06 – 0.10)* | *0.61* |
|  |  |  |
| **Total carbohydrates** | 0.045  (-0.109 – 0.20) | 0.57 |
| *Parity* | *0.28 (0.22-0.34)* | *<0.001* |
| *Maternal BMI* | *0.04 (0.02-0.05)* | *<0.001* |
| *Maternal age* | *-0.007 (-0.02- 0.006)* | *0.30* |
| *Alcohol user* | *-0.07 (-0.19- 0.05)* | *0.25* |
| *Smoking* | *0.20 (0.06-0.35)* | *0.005* |
| *Sex of the child* | *-0.35 (-0.44- -0.26)* | *<0.001* |
| *Maternal ethnicity* | *-0.03 (-0.06 – 0.008)* | *0.13* |
| *Education* | *0.09 (0.006-0.16)* | *0.04* |
| *Urbanization level* | *0.009 (-0.02-0.04)* | *0.52* |
| *Total fat (adj. for kcal)* | *0.06 (-0.08-0.20)* | *0.42* |
| *Total protein (adj. for kcal)* | *0.02 (-0.08 – 0.20)* | *0.42* |
|  |  |  |
| **Mono- and disaccharides** | 0.030  (-0.058 – 0.12) | 0.51 |
| *Parity* | *0.28 (0.22-0.34)* | *<0.001* |
| *Maternal BMI* | *0.04 (0.02-0.05)* | *<0.001* |
| *Maternal age* | *-0.007 (-0.03-0.006)* | *0.27* |
| *Alcohol user* | *-0.06 (-0.17-0.06)* | *0.32* |
| *Smoking* | *0.19 (0.05-0.33)* | *0.009* |
| *Sex of the child* | *-0.35 (-0.44 - -0.26)* | *<0.001* |
| *Maternal ethnicity* | *-0.03 (-0.06 – 0.008)* | *0.12* |
| *Education* | *0.07 (-0.005 – 0.15)* | *0.07* |
| *Urbanization level* | *0.01 (-0.02-0.04)* | *0.46* |
| *Total fat (adj. for kcal)* | *0.06 (-0.02-0.13)* | *0.17* |
| *Total protein (adj. for kcal)* | *0.01 (-0.05-0.07)* | *0.74* |
| *Polysaccharides (adj. for kcal)* | *0.08 (0.008-0.14)* | *0.03* |
|  |  |  |
| **Polysaccharides** | **0.076**  **(0.008 - 0.144)** | **0.03** |
| *Parity* | *0.28 (0.22-0.34)* | *<0.001* |
| *Maternal BMI* | *0.04 (0.03-0.05)* | *<0.001* |
| *Maternal age* | *-0.007 (-0.02-0.006)* | *0.27* |
| *Alcohol user* | *-0.06 (-0.17 – 0.06)* | *0.32* |
| *Smoking* | *0.19 (0.05-0.33)* | *0.009* |
| *Sex of the child* | *-0.35 (-0.44- -0.26)* | *<0.001* |
| *Maternal ethnicity* | *-0.03 (-0.06-0.008)* | *0.12* |
| *Education* | *0.07 (-0.005 – 0.15)* | *0.07* |
| *Urbanization level* | *0.01 (-0.02-0.04)* | *0.46* |
| *Total fat (adj. for kcal)* | *0.06 (-0.02 – 0.13)* | *0.17* |
| *Total protein (adj. for kcal)* | *0.01 (-0.05-0.07)* | *0.74* |
| *Mono- and disaccharides (adj. for kcal)* | *0.03 (-0.06 – 0.12)* | *0.51* |
